# Management of advanced HR-positive breast cancer using metabolically supported chemotherapy and repurposed drugs: a case report

**DOI:** 10.3389/fonc.2026.1795402

**Published:** 2026-04-22

**Authors:** Abdul Kadir Slocum, Didem Tastekin, Tomas Duraj, Thomas N. Seyfried

**Affiliations:** 1 Oncology, ChemoThermia Oncology Center, Istanbul, Türkiye; 2 Oncology, Biruni Universitesi Tip Fakultesi, Istanbul, Türkiye; 3 Biology, Boston College, Boston, MA, United States

**Keywords:** breast cancer, hyperbaric oxygen therapy, hyperthermia, ketogenic diet, metabolic therapy, metabolically supported chemotherapy, repurposed drugs

## Abstract

**Introduction:**

Metastatic hormone receptor-positive (HR+) breast cancer is largely incurable once resistance to conventional treatments occurs. Emerging evidence suggests that progression free and overall survival can improve by targeting the distinct metabolic phenotype of cancer cells (Warburg effect). We report a durable response in a patient with advanced metastatic breast cancer treated with a multimodal “press-pulse” metabolic strategy.

**Case presentation:**

A 49-year-old female from Torino, Italy presented with Stage IV (cT4N1M1) invasive ductal carcinoma (HR+/HER2-, grade 3) with extensive osseous and lymph node metastases, poor performance status (ECOG 3) and severe, debilitating pain. She underwent a combinatorial protocol at ChemoThermia Oncology Center (Istanbul, Turkey) comprising of Metabolically Supported Chemotherapy (MSCT) consisting of docetaxel, doxorubicin, and cyclophosphamide administered following a 14-hour fast and low dose insulin-induced mild hypoglycemia, alongside a strict ketogenic diet (GKI < 2.0). Adjunctive therapies included local and whole-body hyperthermia, hyperbaric oxygen therapy (HBOT), and a combination of repurposed drugs (metformin, aspirin, doxycycline, mebendazole, ivermectin, and famotidine) designed to target metabolic, inflammatory, and survival pathways.

**Results:**

This multimodal treatment protocol was well tolerated, and grade 3/4 adverse events were not observed. The patient noticed symptomatic improvement and functional recovery shortly following the onset of therapy. Follow-up PET-CT scan conducted at 3 months revealed reduced tumor burden. At 6 months, the patient was reported to have a near complete response with the resolution of active bone metastases. On a maintenance schedule, the patient remains in sustained remission as of January 2026, over three years following diagnosis, with a full return to normal daily activities (ECOG 0).

**Conclusion:**

This case highlights the potential of a comprehensive metabolic approach to cancer treatment that combines therapeutic ketosis, metabolically supported chemotherapy, physical modalities (hyperthermia/HBOT), and repurposed drugs. A durable response in a patient with otherwise poor prognosis was achieved after systematically targeting cancer cell bioenergetics and the tumor microenvironment. These findings support further clinical investigation into multimodal metabolic therapies for advanced HR+ breast cancer.

## Introduction

Breast cancer remains one of the most common malignancies and a leading cause of cancer death worldwide. The latest global cancer statistics show that breast cancer in females constituted about 2.3 million new cancer cases and 666,000 deaths in 2022 (1). Breast cancer consists of different subtypes depending on the expression of hormone receptors (estrogen and/or progesterone), human epidermal growth factor receptor 2 (HER2), and the lack of such antigens (triple-negative). Notably, most breast cancers are hormone receptor-positive (HR+). Although such neoplastic diseases are highly sensitive to hormonal therapy, patients with advanced and/or metastatic HR+/luminal breast cancers inevitably become refractory. Despite recent therapeutic advances (e.g. CDK4/6 inhibitors) that have extended survival (2), HR+ breast cancer with extensive metastasis remains essentially incurable, with a median overall survival of only a few years (3). Thus, there is an urgent demand for new novel therapeutic approaches, especially when conventional treatment options have been exhausted.

The conventional management of malignant breast cancer is driven by cancer subtype and stage, with a combination of surgery, radiotherapy, systemic endocrine therapy for HR+ disease, HER2-directed therapies for HER2+ cancers, and chemotherapy. Endocrine therapy, such as aromatase inhibitors or selective estrogen receptor modulators, forms the foundation for HR+ breast cancer, frequently combined with targeted therapies like CDK4/6 inhibitors in metastatic disease. These interventions can prolong disease control, but resistance invariably emerges (4). Once tumors become refractory to hormonal therapy, treatment options are limited to sequential chemotherapies which confer diminishing benefits and cumulative toxicities. As a result, there is growing interest in metabolism-based therapies and repurposed drug approaches that target cancer cell vulnerabilities beyond those addressed by standard cytotoxic or hormonal agents.

An emerging therapeutic strategy is to target the distinct metabolic phenotype of cancer cells. All major cancers, including breast cancer, exhibit a dependency on elevated cytosolic substrate-level phosphorylation (glycolysis) to drive both biomass synthesis and oxygen-independent ATP production (5–7). This metabolic reprogramming is associated with mitochondrial dysfunction (8). Consequently, strategies that restrict glucose availability or force a reliance on oxidative metabolism can selectively pressure cancer cells while sparing normal cells. Calorie restriction and ketogenic diets (KD) are being explored in this context (9). Nutritionally balanced diets that are high in fats and very low in carbohydrates will lower circulating glucose and insulin levels while elevating circulating ketone bodies (β-hydroxybutyrate and acetoacetate) thus inducing a state of nutritional ketosis. The glucose ketone index (GKI) was developed as a quantitative blood biomarker for assessing the state of therapeutic ketosis where values of 2.0 or below induce metabolic stress on tumor cells (10, 11). Normal cells can adapt by utilizing ketone bodies and fatty acids as alternative fuels to glucose, but cancers exhibit reduced ketolytic capacity due to insufficiency of OxPhos linked to mitochondrial dysfunction (11–13). Preclinical studies and early clinical trials indicate that ketogenic metabolic therapy may slow tumor growth and enhance the effects of other cytotoxic treatments (14, 15). Fasting or fasting-mimicking diets can have similar effects; even short-term fasting has been shown to reduce blood glucose and insulin-like growth factor-1 (IGF-1) levels, which are critical growth factors for cancer cells (16). Such metabolic interventions create an unfavorable environment for cancer cells that induce cellular stress responses like autophagy, making tumor cells more susceptible to cytotoxic therapy. However, while early data is promising, the broader clinical evidence surrounding standalone ketogenic diets in cancer remains mixed; such inconsistencies may stem from dietary formulations that fail to adequately and consistently lower GKI values into a therapeutic range, or from the concurrent use of treatments that have not been temporally or mechanistically adjusted to synergize with metabolic interventions (e.g., corticosteroids) (17, 18).

Building on these insights, we developed metabolically-supported chemotherapy (MSCT) protocols to enhance treatment efficacy. MSCT refers to the use of the patient’s metabolic condition to exploit cancer cell vulnerability. In our case, MSCT specifically involves fasting for 14 hours and low-dose insulin potentiation therapy (19, 20), which are performed prior to chemotherapy. Cancer cells, which are highly dependent on glucose, experience acute metabolic stress under these conditions, potentially increasing their uptake of chemotherapeutic agents and amplifying drug-induced cytotoxicity. This concept—described as a “press-pulse” therapeutic strategy—has shown encouraging results in several cancers (11). For example, a recent publication in patients with metastatic lung cancer found that combining a ketogenic diet with MSCT significantly improved response rates and survival compared to standard of care outcomes (21). Similarly, other case reports and case series in advanced cancers have documented significantly improved responses using protocols that integrate fasting, low dose insulin-induced mild hypoglycemia, chemotherapy, ketogenic diet, hyperthermia and hyperbaric oxygen therapy (21–28). A ketogenic diet is often maintained during therapy to induce a relative reduction in glycolytic fuel supply and insulin signaling to the tumor. Collectively, these interventions aim to exploit the metabolic flexibility of normal cells vs. the metabolic inflexibility of cancer cells, thereby sensitizing the tumor to treatment while protecting healthy tissues (11).

Other metabolic therapies may be integrated into the MSCT and KD combination to increase stress to cancer cells. Hyperthermia (HT), the therapeutic application of heat, exploits the fact that cancer cells are more sensitive to high temperatures than normal cells. Local or regional HT (raising tumor temperature to ~42 °C) can damage cancer cell proteins and membranes, induce direct cell death, and improve perfusion, thereby enhancing delivery of oxygen and drugs to the tumor. Clinically, HT can synergize with chemotherapy and radiotherapy, increasing tumor response rates in several trials (29). Hyperbaric oxygen therapy (HBOT) is another modality used to counteract tumor hypoxia, a common feature of aggressive cancers that contributes to treatment resistance. By having patients breathe high concentration oxygen under elevated atmospheric pressure, HBOT significantly increases the amount of dissolved oxygen in the blood and tissues (15, 30). Elevated oxygen tension in the tumor microenvironment can generate reactive oxygen species (ROS) in hypoxic cancer cells and re-sensitize tumors to therapies like radiation or chemotherapy. Indeed, HBOT is an effective method for overcoming tumor hypoxia and has been shown to improve drug penetration and radiation response in preclinical models (31, 32). Neither hyperthermia nor HBOT used alone are curative, but as adjuncts they target the vulnerabilities of cancer cells (heat sensitivity and tumor hypoxia, respectively) and have demonstrated safety and feasibility in integrative treatment protocols.

In addition to dietary and physical therapies, there is growing interest in repurposing well-known non-oncology drugs as anticancer adjuvants. Metformin, one of the first-line oral therapies for type 2 diabetes treatment, has been related to improved cancer outcomes in observational studies. In preclinical models and clinical data, metformin acts as an activator of AMP-activated protein kinase (AMPK) and a mitochondrial complex I inhibitor in the liver, which can suppress the PI3K/Akt/mTOR signaling pathway and lower blood insulin and glucose concentrations, creating unfavorable conditions for tumor growth (33). Aspirin is a widely used nonsteroidal anti-inflammatory medication (NSAID) that has been recognized to exhibit good anticancer properties in preclinical *in vitro* and *in vivo* studies (34). At low doses, aspirin’s antiplatelet activity may prevent circulating tumor cells from evading immune surveillance and forming metastases while high-dose aspirin exerts inhibitory effects on cyclooxygenase-2 (COX-2) and the PI3K/Akt oncogenic pathway. A comprehensive 2025 meta-analysis confirmed that regular post-diagnostic aspirin use is associated with a significant reduction in breast cancer-specific mortality rate by 23% compared with controls (34). Another repurposed drug, doxycycline, is a tetracycline antibiotic that reaches high intratumoral concentrations and inhibits mitochondrial protein synthesis. In cell culture studies, doxycycline has been shown to decrease cancer cell invasive potential by downregulating matrix metalloproteinases (MMP-2/9) and reverse epithelial-to-mesenchymal transition (EMT) markers in various cancers (35). In preclinical *in vivo* murine breast cancer models, doxycycline decreased the tumor burden in bone metastases with reduced proliferation indices, especially in combination with bone-targeted therapies (36). Recent studies show that the antiparasitic drug, mebendazole, has inhibitory effects on both glucose-driven glycolysis and glutamine-driven glutaminolysis, the two metabolic pathways necessary and sufficient for tumor cell proliferation (37, 38). Mebendazole has broad anti-tumor activity across cancer types; notably, it inhibited metastatic spread in preclinical thyroid and colon cancer models and produced regression of lung and lymph node metastases in refractory colorectal cancer in case studies (39). Another antiparasitic, ivermectin, has shown multiple mechanism-oriented activities in cancer treatment. *In vitro*, ivermectin inhibits the Wnt/β-catenin pathways with downregulation in oncogenic cyclin D1 in cancerous cells (40). It also inhibits the PAK1 kinase and AKT/mTOR signaling, which are involved in cancer cell proliferation and drug resistance, including in breast cancer (40). Recent research in endocrine-resistant breast cancer cell lines showed that ivermectin can suppress invasiveness by inhibiting Wnt pathway components, effectively slowing the progression of hormonally resistant tumor cells (40). Finally, H2 histamine receptor antagonists, such as famotidine, show potential in modulating immune response in cancer treatment. Tumor-derived histamine acting on H2 receptors can dampen T-cell and natural killer (NK) cell activity, so blocking these receptors may enhance anti-tumor immunity. In fact, early clinical studies found that short-term famotidine treatment before surgery increased tumor-infiltrating lymphocytes in breast tumors, and patients who developed robust lymphocytic infiltration showed improved disease-free survival (41). This suggests H2 blockers might improve immune responses against cancer when used as adjuncts.

Individually and in combination, these metabolic therapies and repurposed drug strategies aim to target multiple hallmarks of cancer (metabolic dysregulation, evasion of cell death, invasiveness, and immune suppression) in a comprehensive manner. There is emerging clinical evidence that suggests that a combination of nutritional ketosis, fasting/insulin-induced mild hypoglycemia before chemotherapy, hyperthermia, HBOT, and repurposed drugs are clinically feasible and may have efficacy when combined with standard of care (21). Based on this rationale, we implemented an integrative protocol for a patient with advanced HR+ breast cancer. This is a report of a single patient that underwent metabolically supported chemotherapy, in combination with a ketogenic diet, hyperthermia, HBOT, and a regimen of repurposed drugs (metformin, aspirin, doxycycline, mebendazole, ivermectin, and famotidine). This multimodal approach was associated with a complete response with three years of disease management in this patient with end-stage breast cancer. The results from this case report may inform further research into metabolic therapies as a complementary strategy for managing advanced breast cancers.

## Case presentation

A 49-year-old woman from Torino, Italy with no significant comorbidities presented in early 2022 with progressively worsening left breast pain, breast deformity, and severe back pain. An MRI done at Lugano, Switzerland in July 2022 revealed a 29 × 25 × 37 mm spiculated mass in the left breast with skin and pectoralis muscle invasion, a 6 mm satellite lesion, and multiple suspicious left axillary lymph nodes; suspected bone metastases were noted in the sternum, clavicles, and ribs. Ultrasound-guided core needle biopsies confirmed an invasive ductal carcinoma (Nottingham grade 3) with associated high-grade ductal carcinoma *in situ*. The tumor was strongly estrogen receptor-positive (>90% cells) and progesterone receptor-positive (~60%), with low HER2 expression (score 1+ by immunohistochemistry) and Ki67 25%. Clinically, the patient’s performance status had deteriorated rapidly; she suffered debilitating bone pain with limited mobility (ECOG 3). Local physicians in Switzerland deemed the cancer inoperable and advised that palliative systemic therapy was feasible. Given the prognosis and lack of curative options, the patient sought an integrated oncologic approach seeking to improve outcomes compared to standard of care alone.

**Figure 1:**
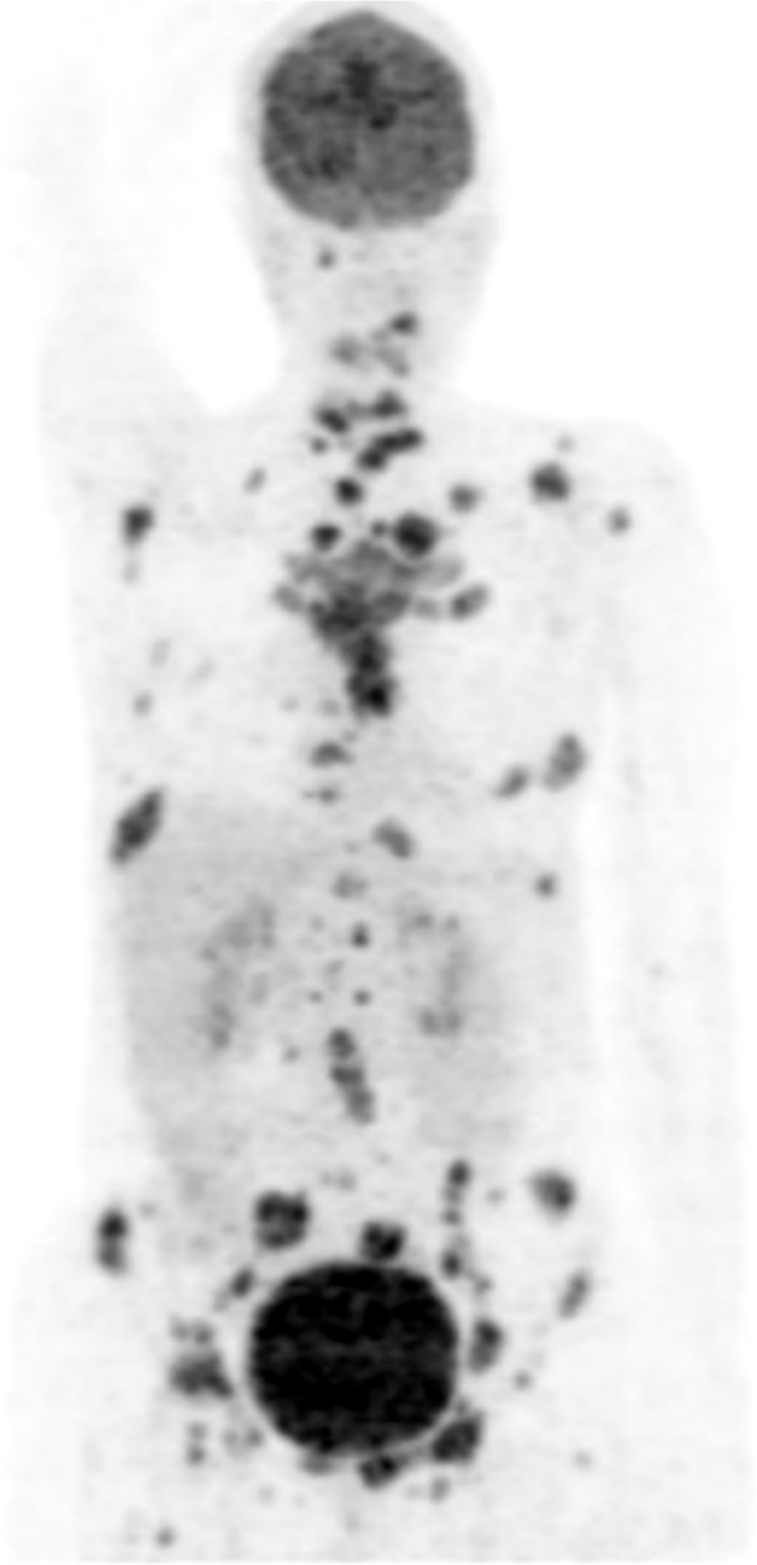
The initial PET-CT (29.08.2022) showed an FDG-avid malignant left breast primary (SUVmax 6.2) in the lower outer quadrant with skin infiltration/retraction, plus an adjacent FDG-avid nodule (SUVmax 3.2) suspicious for a satellite lesion. FDG-avid left axillary level I nodes and a left parasternal FDG-avid focus (SUVmax 4.6) were suspicious for metastatic lymphadenopathy, and a small non–FDG-avid left pleural effusion. There was extensive FDG-avid (SUVmax 11.2) lytic metastatic involvement throughout the skeleton (skull, spine, ribs, sternum, clavicles, scapulae, pelvis, proximal femurs, and proximal left humerus).

In August 2022, she was evaluated at ChemoThermia Oncology Center (Istanbul,Turkey). Informed consent was obtained for both diagnostic and therapeutic interventions. A staging 18F-FDG PET/CT demonstrated an FDG-avid primary tumor in the left breast (SUVmax 6.2) with an adjacent malignant satellite nodule (SUVmax 3.2), FDG uptake in a few axillary lymph nodes, and extensive osseous metastases involving the calvarium, multiple foci in the vertebral column, bilateral scapulae, clavicles, sternum, some ribs of bilateral hemithorax, bilateral pelvic bones, proximal parts of bilateral femur and the proximal part of the left humerus (SUVmax 11.2). No visceral metastases were identified (**Figure 1**). The disease was therefore characterized as stage IV (cT4N1M1) hormone receptor-positive, HER2-negative breast cancer. Her detailed laboratory tests performed during onboarding reported Alkaline phosphatase (ALP) 352 U/L, Lactate dehydrogenase (LDH) 339 U/L, C reactive protein (CRP) 30.43 mg/L, Sedimentation (1 hour) 58.00 mm/hour, Carcinoembryonic antigen (CEA) 7.17 ng/mL and Cancer antigen 15-3 (CA15-3) 218.60 U/mL.

**Figure 2:**
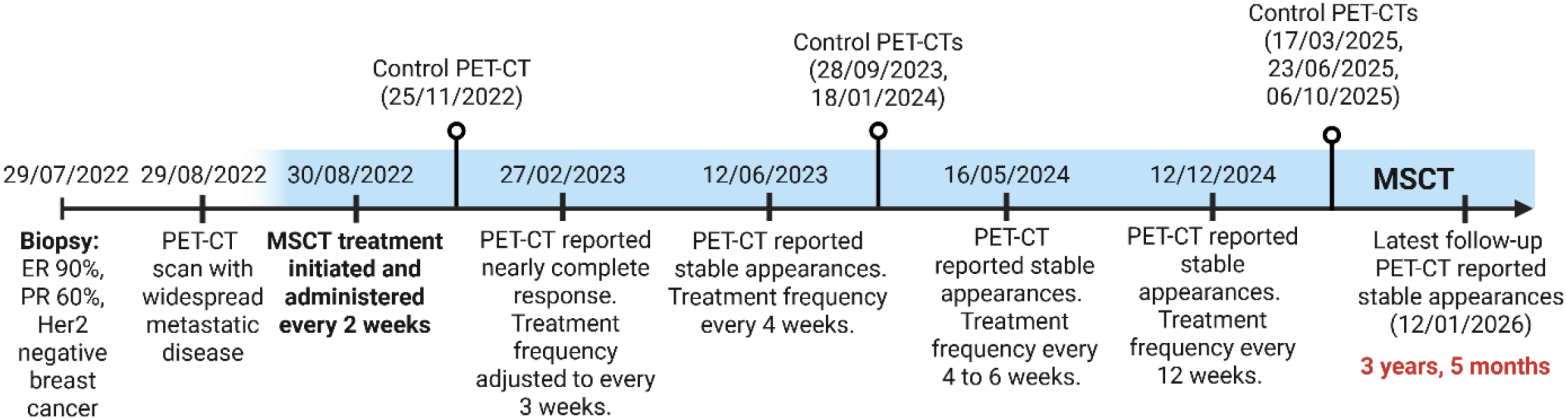
Treatment timeline. Metabolically supported chemotherapy (MSCT) was initiated 1 month after the diagnosis and maintained at gradually longer frequency intervals with follow-up PET-CT scans to date indicating stable radiologic appearances.

**Figure 1** subsequently, an intensive integrative treatment regimen was implemented, comprising metabolically supported chemotherapy (MSCT) with the TAC regimen (docetaxel 30 mg/m2, doxorubicin 20 mg/m2, and cyclophosphamide 250 mg/m2) being administered in a *press-pulse* fashion, in combination with a ketogenic diet (KD), hyperthermia (local & whole body), hyperbaric oxygen therapy (HBOT), as well as the use of several repurposed drugs (**Figure 2**).

### Metabolically supported chemotherapy

Premedication consisted of 45.5 mg pheniramine maleate, 0.25mg palonosetron HCl and regular insulin (Humulin R) in doses ranging between 1 to 5 IU (in order to achieve a state of mild asymptomatic hypoglycemia with blood glucose levels around 50–60 mg/dl in accordance with published MSCT protocols) (21–28).

The patient visited our clinic for treatment sessions following a minimum 14 hour of fast, and her blood glucose level was measured upon admission. Then this level was down-titrated to the targeted pretreatment mild hypoglycemia (blood glucose ~50–60 mg/dL) level with low dose insulin (1 to 5 IU) administration. This protocol, based on prior reports, aims to exploit cancer cells’ dependence on glucose and enhance chemo-sensitivity during the window of insulin-induced mild hypoglycemia. She was closely monitored for hypoglycemia signs/symptoms and blood glucose levels by the attending physician and nurse. An IV line for dextrose administration was always kept open. No adverse events were encountered due to fasting and/or hypoglycemia. She received a chemotherapy regimen consisting of docetaxel (30 mg/m2), doxorubicin (20 mg/m2), and cyclophosphamide (250 mg/m2). The patient received this combination chemotherapy regimen on a 2-week cycle. Zoledronic acid 4 mg was given every 28 days to manage metastatic bone disease.

### Therapeutic ketosis

The patient was started on a strict ketogenic diet under close supervision of the clinician and has continued it uninterrupted after initial MSCT cycles. The diet followed a classical ketogenic macronutrient distribution (approximately 70–80% of total calories from fat, 15–20% from protein, and <10% from carbohydrates), with daily carbohydrate intake restricted to less than 20 grams. Her meals consisted predominantly of olive oil, avocado, nuts, seeds, eggs, moderate portions of fish and meat, and low-carbohydrate vegetables, while grains, sugars, starchy foods, and fruit were eliminated. Capillary blood glucose, HbA1C and β-hydroxybutyrate levels were monitored regularly, both during routine days and around treatment sessions, to assess metabolic compliance. The patient’s GKI during inpatient visits ranged between 3.8 to 2.6 during the first 3 months of treatment, 2.9 to 1.8 during the second 3 months of treatment and has been consistently below 2 in subsequent cycles. Her HbA1C ranged below 5.2 with the most recent result from January 2026 being 4.3. Under this regimen, the patient consistently achieved and maintained a state of therapeutic ketosis, with no reported dietary lapses, and experienced only mild, transient side effects (such as fatigue and constipation) that were managed conservatively. Her body weight and performance status remained stable or improved over time, and the ketogenic diet was continued as a core component of the metabolic protocol during both the initial and maintenance phases of treatment.

### Hyperthermia

Local hyperthermia was administered as 60-minute sessions using a modulated electro-hyperthermia device (OncoTherm EHY-3010, OncoTherm, Troisdorf, Germany). This capacitive radiofrequency system (13.56 MHz) selectively couples energy into malignant tissue based on impedance differences between tumor and surrounding normal tissues, thereby increasing “metabolic pressure” on cancer cells while minimizing heating of the overlying skin and adjacent healthy structures. A 40 × 50 cm flexible textile electrode was positioned from the supraclavicular region over the anterior thorax and upper abdomen, encompassing the primary breast lesion, regional nodal basins, bilateral ribs, shoulders and some of the osseous metastases. Energy was escalated stepwise to achieve deep regional heating, with the aim of reaching cytotoxic intratumoral temperatures above 43 °C and increasing local blood flow to enhance delivery of administered chemotherapy. Direct intratumoral temperature measurements were not performed; instead, tissue temperatures were estimated indirectly from applied power and impedance, with calculated peak tumor temperatures for this patient’s sessions ranging between approximately 43.8 °C and 45.1 °C. The treatments were well tolerated, with only mild, transient erythema in the treatment field and no clinically relevant skin burns.

In addition to local hyperthermia treatment, the patient underwent 150-minute sessions of fever-range whole-body hyperthermia using a Heckel HT-3000 system (Hydrosun Medizintechnik GmbH, Germany). The device employs water-filtered infrared-A (wIRA) radiators mounted above an insulated treatment tent to raise the patient’s core temperature in a controlled and well-tolerated manner while the patient rests on an adjustable treatment bed. During these sessions, the patient’s core temperature was gradually increased from normothermia to a therapeutic fever range (approximately 38.5 – 39.5 °C) and maintained for a defined period under continuous monitoring of vital signs and comfort. Whole-body hyperthermia was used as a systemic adjunct to the overall metabolic protocol, with the intent of improving global perfusion, enhancing chemosensitivity, and supporting antitumor immune responses, in line with contemporary concepts of hyperthermia as a sensitizer for chemotherapy, radiotherapy and immunotherapy (42).

### Hyperbaric oxygen therapy

Hyperbaric oxygen therapy (HBOT) was delivered in 60-minute sessions using a portable, soft-shelled monoplace chamber (Quamvis 320^®^, OxyHealth, Santa Fe Springs, CA, USA). This chamber has a rigid external frame and a spacious 32-inch internal diameter, allowing the patient to lie supine with good visibility and easy accessibility for entry and exit. The system was pressurized with an oil-free clean-air compressor equipped with high-efficiency inline filtration capable of removing particles down to approximately 0.01 µm, and connected to an oxygen concentrator providing a continuous flow of medical-grade oxygen (≈10 L/min) into the breathing circuit. Each treatment aimed to achieve a mild hyperbaric environment at 1.5 ATA, during which the patient breathed high-concentration oxygen inside the pressurized chamber to increase tumor oxygenation, counteract hypoxia, and potentiate therapy effects. Throughout the sessions, vital signs and subjective tolerance were monitored; the patient did not experience barotrauma, significant ear discomfort, or claustrophobic reactions, and no treatment-limiting adverse events occurred.

The patient was planned to receive treatments on 3 day cycles, where day 1 included all therapies (metabolically supported chemotherapy, whole-body hyperthermia, local hyperthermia, HBOT) and day 2 and 3 included local hyperthermia and HBOT. The patient completed all planned local and whole-body hyperthermia and HBOT sessions without any adverse events higher than grade 1 (CTCAE).

### Repurposed drugs

Alongside these modalities, a combination of six repurposed non-oncology drugs was introduced based on the emerging evidence for synergistic effects against cancer. These included metformin (1.000 mg/day) for gluconeogenesis inhibition; aspirin (100 mg/day), for its antiplatelet and anti-inflammatory effects; doxycycline (100 mg/day), an antibiotic that targets mitochondria and stem-like cancer cells; mebendazole (100 mg/day), an anthelmintic that disrupts microtubules and induces apoptosis in cancer cells; ivermectin (6 mg/day), which can block Wnt/β-catenin and AKT/mTOR signaling, suppressing proliferation and EMT in breast cancer; and famotidine (40 mg/day), an H2 blocker used to enhance anti-tumor immunity by inhibiting tumor-associated immunosuppressive histamine signaling. The rationale behind this treatment was for simultaneous targeting of cancer metabolism, inflammation, invasiveness, and immune evasion using FDA/EMA-approved drugs that are well tolerated. Repurposed drugs were used at the specified dosages continuously, based on the scientific rationale and previous clinical experience at our institution. The patient was monitored closely for drug-drug interactions. No drug interactions were observed, and the patient did not present any adverse events higher than grade 1.

#### Treatment course and response

The patient’s tolerance of therapy was excellent, with no significant acute toxicities observed. Notably, she experienced rapid symptomatic relief. After the first cycle of MSCT with adjunctive metabolic therapies, her intractable bone pain markedly diminished, improving sleep quality. After the second cycle of treatment, additional improvement occurred, as the patient was able to lie flat on the bed when previously impaired due to pain. The patient was also able to stand with support, whereas the patient was bedridden before the onset of treatment. By the second month of the treatment, the patient showed improvement in motor functions, being able to walk independently for short distances. Aside from minor hair loss, nausea and fatigue around treatment days, she had no serious side effects. Her quality of life improved significantly as pain subsided and mobility returned.

**Figure 3:**
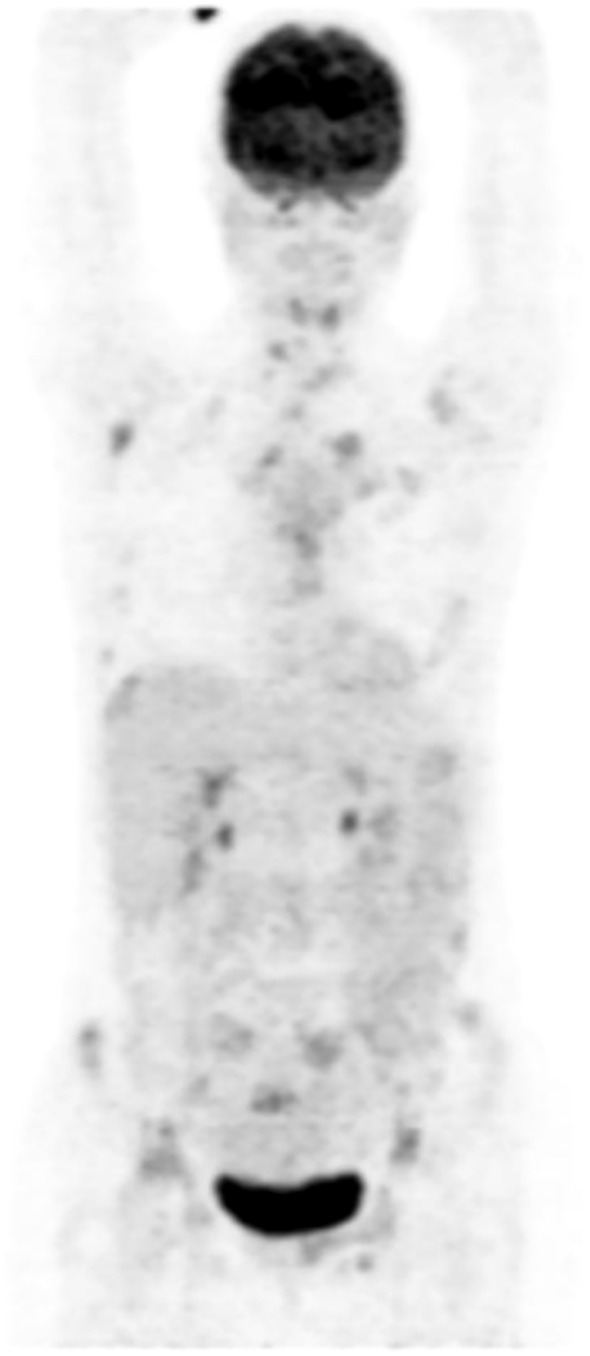
PET-CT evaluation at 3 months of therapy (25.11.2022). Compared with the PET-CT from 29.08.2022, the FDG-avid left breast lesion has markedly regressed (current SUVmax 2; previous SUVmax 6.2) with only minimal–mild residual uptake, and the previously suspicious adjacent nodule is now millimetric and FDG-negative. Left axillary nodes have regressed, the prior small left pleural effusion has completely resolved, and the widespread skeletal metastases show major metabolic regression (current SUV max 5.2; previous SUVmax 11.2) with sclerotic change consistent with treatment response.

A response evaluation scan was performed at about 3 months into therapy. A PET/CT scan performed in late November 2022 (compared to baseline August 2022) showed tumor regression: the primary breast lesion had shrunk clinically and had nearly no metabolic activity (SUVmax reduced from 6.2 to ~2.0), and the previously noted satellite nodule had become metabolically inactive and regressed to a small residual on imaging. The faint FDG uptake in a left axillary lymph node seen on baseline scan had resolved, with no new lymphadenopathy. All distant metastases showed remarkable improvement. Numerous lytic bone lesions exhibited sclerosis on CT and a dramatic reduction in FDG uptake (for example, SUVmax of the most active bone metastasis reduced from 11.2 to 5.2). No new lesions emerged, and a small pleural effusion noted at baseline had completely disappeared (**Figure 3**). Given this good interim response, her treatment was decided to be continued with the same protocol and frequency of treatment (**Figure 3**).

**Figure 4:**
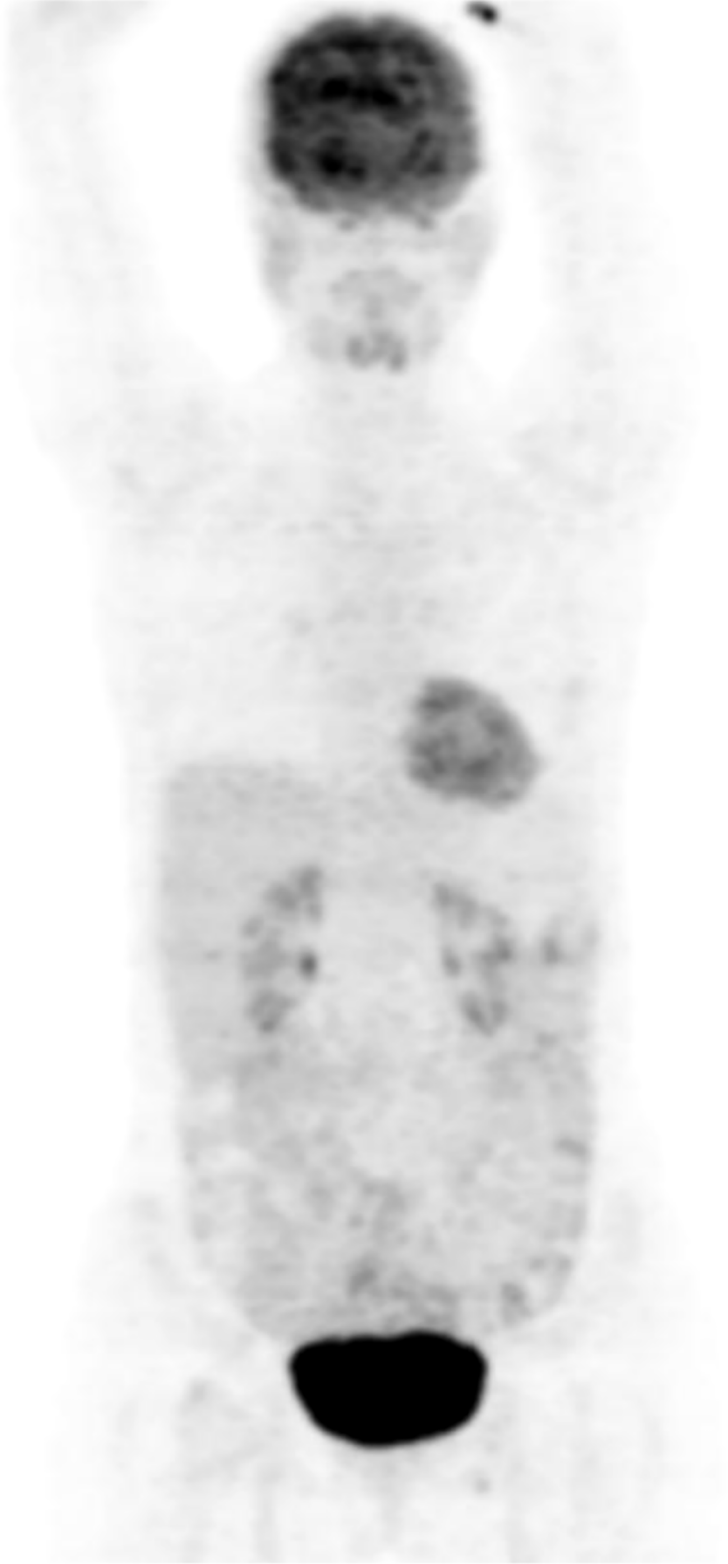
PET-CT evaluation at 6 months of therapy (27.02.2023). Compared with the prior PET-CT, there is stable minimal FDG uptake (SUVmax 1.9) at the left breast postoperative suture site without significant change. Previously noted small left axillary level I nodes now show no significant FDG uptake, and metastatic sclerotic bone lesions demonstrate metabolic near-complete regression with only minimal residual uptake in some sites (SUVmax 2.9). Overall, the findings are consistent with a nearly complete treatment response.

On her second response evaluation scan done at about 6 months into therapy, the patient demonstrated a nearly complete response to therapy. A follow-up PET/CT scan in late February 2023 demonstrated overall metabolic complete regression of the previously observed multiple sclerotic bone metastases. Only very minimal residual FDG uptake remained in a few lesions, most prominently in the sacral vertebrae (SUVmax 2.9) (**Figure 4**).

In addition to the near-complete radiologic response, the patient’s performance status improved to ECOG 0-1, accompanied by pain resolution and a return to normal daily activities (**Figure 4**).

#### Long-term outcome

Following the initial 6 months of therapy, the patient’s treatment interval was gradually extended initially to in-clinic visits for treatment (MSCT, hyperthermia and HBOT) every 3 weeks for the following 3 months. With the follow-up scan reporting stable appearances with near complete response to treatment, the interval was increased to every 4 weeks, with the addition of capecitabine 1.500 mg/day and tamoxifen 20 mg/day. Her treatment was maintained for 12 months at this schedule and as scans reported stable appearances, therapy frequency was changed to treatment cycle intervals being every 4 to 6 weeks during the subsequent 6 months, with reduction of capecitabine to 1.000 mg/day. Subsequently, as scans again reported stable appearances, treatment cycle intervals were further extended to every 12 weeks in December 2024.

**Figure 5:**
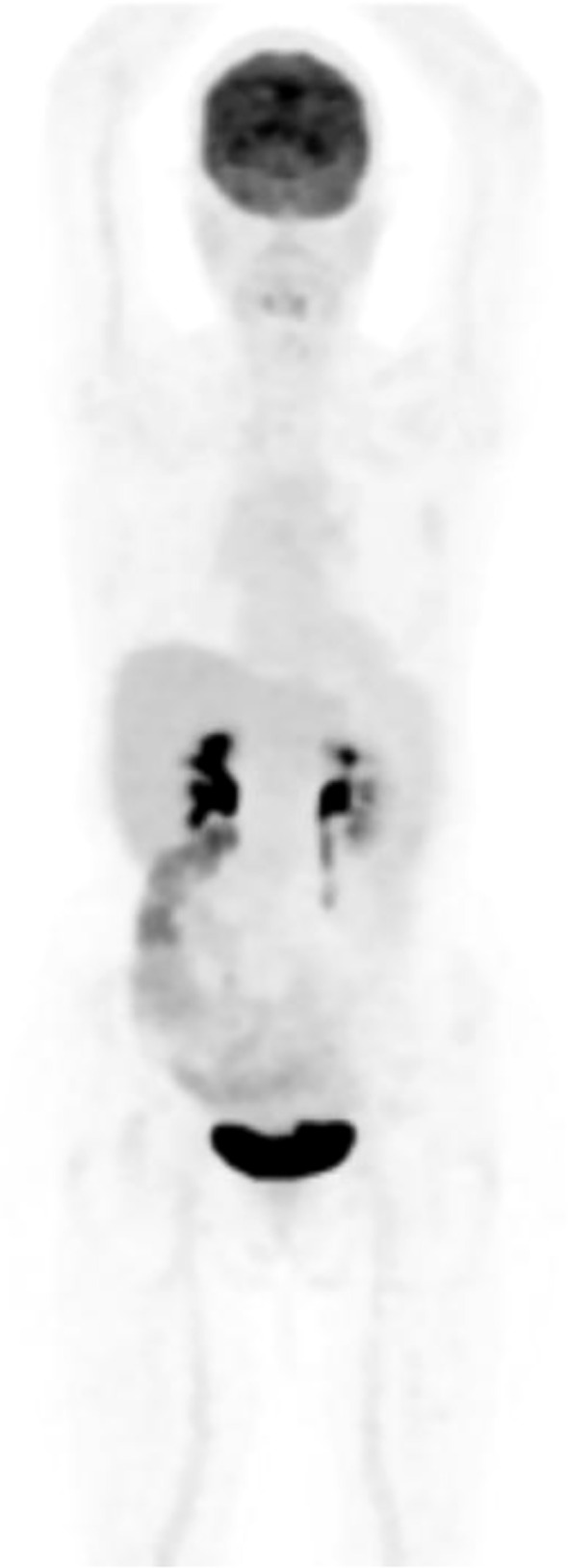
Latest follow-up PET-CT (13.01.2026). No distinct FDG-avid hypermetabolic focus is seen in the remaining left breast parenchyma to suggest residual or recurrent primary disease. Numerous sclerotic metastatic bone lesions persist with a similar appearance to the prior study, with only minimal hypermetabolism in some areas.

During this maintenance phase, she remained on the ketogenic diet and continued oral metformin, aspirin, doxycycline, mebendazole, ivermectin, and famotidine without interruption. Routine surveillance scans and labs were obtained at regular intervals, all of which showed stable disease with no signs of progression. As of the most recent follow-up PET-CT in January 2026 (around three and a half years since diagnosis), the patient is alive and in sustained remission (**Figure 5**). Laboratory tests done during this PET-CT follow-up visit reported her Alkaline phosphatase (ALP) 45 U/L, Lactate dehydrogenase (LDH) 107 U/L, C reactive protein (CRP) < 2.0 mg/L, Sedimentation (1 hour) 6.00 mm/hour, Carcinoembryonic antigen (CEA) 1.98 ng/mL and Cancer antigen 15-3 (CA15-3) 11.20 U/mL. The patient did not require analgesics for pain management since her initial response, and her mobility and functional status are fully restored. She reports a high quality of life (ECOG 0) and has been able to resume normal activities, living independently and engaging in daily exercise (long walks) (**Figure 5**).

This patient’s case thus represents a sustained durable response in metastatic HR+ breast cancer. No cancer progression has occurred since the initiation of this multi-modal treatment, and the patient remains under close observation while continuing metabolic and repurposed drug maintenance strategies. This outcome is especially noteworthy given that metastatic HR+ breast cancer is generally considered incurable with standard therapies, which typically yield a median progression-free interval of only several months and a 5-year survival rate under 30% in this setting (43). The remarkable response and ongoing disease control observed in this case highlights the potential impact of a multimodal metabolic treatment paradigm for end-stage breast cancer.

## Discussion

We report a durable response to a multimodal metabolic treatment protocol in a patient with metastatic hormone receptor-positive (HR+) breast cancer. The approach combined metabolically supported chemotherapy (MSCT), a calorie-restricted ketogenic diet (KD), local and whole-body hyperthermia, hyperbaric oxygen therapy (HBOT), and a combinatory regimen of repurposed drugs. Taken together, these interventions targeted the metabolic vulnerabilities, microenvironmental factors, and survival pathways characteristic of advanced HR+ disease. The sustained radiologic and metabolic response, along with the improvement in performance status and quality of life, suggests that such a strategy may represent a valuable adjunct in carefully selected patients with end-stage breast cancer.

Breast cancer, including HR+ subtypes, exhibits the metabolic phenotype of cancer originally described by Otto Warburg: aerobic glycolysis with lactic acid production (Warburg effect), increased glucose uptake, and evidence of mitochondrial dysfunction on molecular and functional imaging (8). This metabolic inflexibility renders tumor cells highly dependent on fermentable fuels such as glucose and glutamine for proliferation and energy (38). By contrast, normal cells retain the capacity to flexibly switch between substrates and can more efficiently oxidize ketone bodies when glucose availability is reduced. It should be noted that while our metabolic explanation is supported by systemic markers and the metabolism-based findings of 18F-FDG PET imaging, direct metabolomic tissue analysis was not performed; rather, this conceptual framework is drawn from extensive literature demonstrating the distinct metabolic vulnerabilities of breast cancer (7).

The therapeutic logic of our protocol is built around exploiting this differential fuel handling. KMT lowers circulating glucose (fermentable fuel) and insulin (growth signaling) while inducing nutritional ketosis. In this state, normal tissues adapt to using fat-derived metabolites such as ketone bodies and fatty acids for energy, whereas cancer cells are unable to substitute the relative reduction in glycolytic flux with ketone body oxidation (11). This creates a systemic environment that is metabolically stressful for tumor cells but comparatively tolerable for healthy tissues. Specifically, a restricted, nutritionally balanced ketogenic diet was selected because it effectively functions as a sustainable, long-term fasting-mimicking diet (44) that safely exploits the dependence of mitochondrially abnormal breast cancer cells on glucose (12, 45, 46), while mitigating the long-term safety and adherence limitations typically associated with unrestricted or unbalanced dietary interventions (47). Superimposed on this chronic “press” is the “pulse” of metabolically supported chemotherapy: each cytotoxic treatment is delivered after an overnight fast and preceded by low-dose insulin to induce mild, transient hypoglycemia. This strategy aims to intensify metabolic stress at the time of drug delivery, increase drug uptake into glucose-avid tumor cells, and potentially improve the therapeutic index of chemotherapy (9, 11, 21–28).

Hyperthermia and HBOT further enhance this metabolic targeting. Local and whole-body hyperthermia is intended to elevate tumor temperature to cytotoxic levels, disrupt protein folding, impair DNA repair, and improve tumor perfusion, which in turn may enhance oxygen and drug delivery. HBOT reverts tumor hypoxia by increasing tissue oxygen tension. Under conditions of low glucose and active ketosis, the reactive oxygen species generated by HBOT may preferentially damage cancer cells with mitochondrial defects, while ketone-adapted normal cells are relatively protected (32). In the present case, the integration of KD, fasting/insulin, hyperthermia, and HBOT may have contributed to creating a more hostile, pro-oxidative, and resource-deprived microenvironment for the tumor, amplifying the effects of chemotherapy.

A critical element in this protocol was the use of several well-characterized non-oncology drugs with emerging anticancer properties. Drug repurposing exploits known safety and pharmacokinetics to target cancer cells in diverse ways (48, 49). In this case, six repurposed drugs from distinct pharmacologic classes –an antidiabetic (metformin), a non-steroidal anti-inflammatory drug (aspirin), an antibiotic (doxycycline), two antiparasitics (mebendazole and ivermectin), and a histamine H_2_-receptor antagonist (famotidine)– were selected to act on tumor metabolism, inflammation, invasion, and the tumor microenvironment in a coordinated manner.

Metformin, a first-line oral antidiabetic drug, was included for its dual systemic and direct cellular effects on tumor metabolism. On the system level, it can decrease hepatic gluconeogenesis and increase sensitivity to insulin in the periphery, thereby lowering blood glucose and insulin/IGF-1 concentration, which are key drivers of proliferation and endocrine resistance in HR+ breast cancer (33). At the cellular level, it can activate AMP-activated protein kinase (AMPK) and inhibit mitochondrial complex I, reduce ATP production, suppress mTOR signaling, and shift cells towards a more catabolic metabolic state. In cancer cells that already operate near the limits of bioenergetic stress, these changes can impair growth and enhance susceptibility to both cytotoxic and metabolic stressors. Observational studies linking the use of metformin to improved survival in breast cancer populations offer further support for its addition to the treatment strategy as an adjuvant agent (50, 51).

Low-dose aspirin was used because of its anti-inflammatory and antiplatelet effects (52). Chronic inflammation promotes carcinogenesis and progression through prostaglandin-mediated signaling, NF-κB activation, and modulation of the tumor microenvironment. Aspirin’s inhibition of cyclooxygenase (COX) reduces prostaglandin E_2_ levels and may attenuate pro-tumor inflammatory pathways. The antiplatelet effect can abrogate the binding between platelets and tumor cells that enables evasion of the immune system, sequestration in vessels, and metastasis. Meta-analysis data that suggests reduction in breast cancer-specific mortality among regular aspirin users supports the rationale for using low-dose aspirin as a low-cost adjunct in selected patients (53).

Doxycycline, a tetracycline antibiotic, was added primarily for its mitochondrial and anti-metastatic properties. Doxycycline inhibits mitochondrial protein synthesis, which can be particularly detrimental to cancer cells that rely on altered mitochondrial function and biogenesis. Preclinical studies indicate that doxycycline can deplete cancer stem-like cell populations and reduce markers associated with resistance to therapy. Additionally, doxycycline reduces the synthesis of matrix metalloproteinases (MMP-2 and MMP-9) and reduces EMT markers, thus lowering invasiveness and metastatic capabilities of cancer cells (35). Doxycycline also reduces bone metastasis burden and cell proliferation rates in preclinical models, which further supports the use of doxycycline in cancer patients showing extensive skeletal involvement (54).

Mebendazole has been chosen because of its microtubule-targeting and proapoptotic properties (39). By binding to β-tubulin and disrupting microtubule polymerization, mebendazole induced mitotic arrest at the G_2_/M checkpoint and triggered apoptotic cell death in *in vitro* analyzes. It has demonstrated broad antitumor activity in preclinical models, including inhibition of angiogenesis and suppression of metastatic dissemination. Case series and pilot clinical trials documenting tumor responses in advanced malignancies treated with mebendazole have stimulated interest in its off-label application, particularly in heavily pretreated or refractory settings (39, 55, 56). Larger clinical trials are ongoing (e.g., NCT03925662). Ivermectin, another antiparasitic, has shown pleiotropic anticancer actions in laboratory studies, including inhibition of P-glycoprotein-mediated drug efflux, modulation of Wnt/β-catenin and Hippo/YAP signaling pathways, interference with cancer stem cell maintenance, and enhancement of reactive oxygen species generation (40). These properties may sensitize tumor cells to chemotherapy and metabolic stress and may also contribute to overcoming multi-drug resistance.

Finally, famotidine, a histamine H_2_-receptor antagonist, was used to modulate the tumor microenvironment. Histamine signaling through H_2_ receptors has been implicated in immune suppression, angiogenesis, and tumor proliferation. Although much of the earlier clinical experience involves cimetidine, H_2_ blockade in general has been associated with improved outcomes in gastrointestinal and breast cancers, potentially through enhancement of antitumor immune responses and reduction of perioperative immunosuppression (41). Within our protocol, famotidine was utilized as a low-toxicity means of influencing host–tumor interactions and supporting immune function.

Taken together, this repurposed drug combination was intentionally constructed to target multiple hallmarks of cancer: dysregulated energy metabolism (metformin), chronic inflammation and platelet-mediated dissemination (aspirin), invasion and metastasis (doxycycline and mebendazole), therapy resistance and stemness (doxycycline and ivermectin), and immune modulation within the tumor microenvironment (famotidine, with indirect contributions from aspirin and ivermectin). When layered on top of nutritional ketosis (ketogenic diet), fasting-based metabolic conditioning, hyperthermia, and HBOT, these agents were expected to amplify metabolic and oxidative stress in tumor cells while preserving an acceptable safety profile. The tolerability of the regimen and the durable clinical response observed in this patient support further systematic evaluation of such multi-drug, multi-target repurposing strategies in advanced HR+ breast cancer.

## Comparison to prior research and future perspectives

Importantly, the durable response observed in this patient following standard cytotoxic and metabolic therapy aligns with similar observational outcomes documented in other case reports and case series involving advanced breast and various other solid tumors (9, 21–28, 57, 58). This case follows directly on our previous publication of an end-stage HR+ (ER+/PR+/HER2-) breast cancer patient treated with a similar combinatorial metabolic protocol (28). In our earlier publication, we described a patient with extensive metastatic involvement (including brain, lung, liver, and bone) who was ineligible for conventional therapy due to poor performance status and end stage disease. Under a regimen combining MSCT, KD, hyperthermia, HBOT, and repurposed drugs, all detectable lesions showed complete response to therapy. This response was subsequently sustained for an extended period using a maintenance protocol of metabolic and pharmacological therapies. This patient is still under disease management following 7 years since therapy at our clinic. The present case, likewise, involving HR+ metastatic disease, reproduces a very similar pattern: near-complete response of metabolically active lesions, durable disease control, and improvement in functional status, despite an initial poor prognosis. Together, these two HR+ case reports suggest that this approach may be reproducible within this biological subtype and not merely an isolated, anecdotal response.

In our previous case study of triple-negative breast cancer (TNBC), we employed the same treatment approach (MSCT along with KD, hyperthermia, and HBOT) and observed complete pathologic and radiologic response in a young patient diagnosed with metastatic TNBC (27). Although TNBC and HR+ breast cancers are clinically distinct, the fact that similar metabolic strategies were associated with durable responses in both subtypes is noteworthy. These findings support the concept that bioenergetic abnormalities may be shared across all major cancers (59, 60). Consequently, the targeting of fundamental metabolic vulnerabilities may have broader applicability across different breast cancer phenotypes.

However, such findings must also be considered in the proper context of caution. Indeed, each of the three cases represent single patients without control arms. The level of evidence of such reporting is inherently prone to specific limitations, such as risks of biased sample selection and difficulties in elucidating a clear contribution of each component of a combinatorial protocol. We cannot currently delineate the individual contributions of each therapy nor confirm whether the interactions are synergistic, additive, or neutral; further studies are required to evaluate which parts produced the greatest effect, though the individual risks of each adjunctive therapy should be weighed against the severe prognosis of the cancer diagnosis itself. We also note that combinatorial regimens to potentiate standard of care are receiving increasing attention in clinical trials, such as the CUSP9 (61), COMBAT (62), gMDACT (63), CLOVA (64), MEMMAT (65), renin-angiotensin modulators (66) and COAST (NCT05036226) trials.

While the ‘press-pulse’ rationale aims to sensitize tumor cells, the TAC regimen itself is a potent cytotoxic therapy capable of inducing responses in treatment-naive advanced breast cancer; thus, the proportional contribution of this cytotoxic ‘pulse’ versus the metabolic ‘press’ remains difficult to isolate. Regarding feasibility and real-world applicability, it is important to acknowledge that the administration of this multimodal regimen was conducted within a highly specialized oncologic facility, which may not be applicable to all oncology centers. Therapeutic protocols at our clinic are highly individualized to the patient’s specific clinical presentation, guided by established institutional frameworks and prior published experiences (21–28). Finally, while our mechanistic explanation focusing on cancer metabolism is grounded in previous research, GKI monitoring and 18F-FDG PET findings, we acknowledge the lack of direct metabolic analysis of the tumor tissue as a limitation. We underscore the necessity of future studies to directly confirm these mechanistic hypotheses at the tumor level through metabolic stratification before and after therapeutic interventions (67).

Nevertheless, despite these issues, we consider noteworthy that each aspect of therapy (mechanistic rationale, radiographic response, and clinical durability) was documented across multiple patients and across multiple disease types. In particular, the repeated observation of long-lasting and durable disease control in end-stage HR+ patients with limited conventional therapeutic options suggests that MSCT, metabolic therapy and repurposed drugs might meaningfully extend survival and quality of life in this patient population.

Future clinical research should focus on prospectively evaluating this multimodal approach in larger cohorts of HR+ metastatic breast cancer patients. Priority areas include: identifying biomarkers that predict sensitivity to metabolic interventions; optimizing the composition, dosing, and timing of repurposed drug combinations; and clarifying how to integrate metabolic therapy with standard endocrine, targeted, and cytotoxic regimens. It is important to note that MSCT is compatible with recent additions to standard of care such as CDK4/6 inhibitors (2), and potential additivity and/or synergy should be evaluated. While all the interventions implemented in this case report have clinical data or approval as monotherapy, controlled trials would be necessary to delineate the relative importance of each component (KD, fasting, MSCT, hyperthermia, HBOT, and single-agent repurposed drugs), as well as to assess feasibility and safety in broader clinical practice.

In summary, this case, together with our previous HR+ and TNBC publications, supports the view that dose-adjusted chemotherapy, cancer metabolism targeting and specific drug repurposing can induce durable responses in patients with otherwise limited therapeutic options. While confirmatory studies in larger cohorts are needed, these findings strengthen the rationale of press-pulse metabolic therapies in breast cancer and justify further clinical investigation of such combinatorial metabolic protocols.

## Conclusions

This case adds to our two prior publications by suggesting that advanced, HR+ metastatic breast cancer may display improved therapeutic outcomes when tumor metabolism and microenvironment are targeted in a systematic and coordinated way. Across all three patients (two with HR+ disease and one with metastatic TNBC), we consistently observed durable tumor regression, recovery of functional capacity, and significant improvement in quality of life under a protocol combining MSCT, nutritional ketosis, local and whole-body hyperthermia, HBOT, and a combination of rationally selected repurposed drugs. The reproducibility of these outcomes in biologically distinct subtypes, all with poor baseline prognosis, suggests that the metabolic vulnerabilities of breast cancer cells can be clinically leveraged within standard oncology practice.

Though these case reports are drawn from individual cases, taken together, they collectively point towards a promising outlook for cancer care. While these findings represent a single patient in a heterogeneous case series, they suggest that metabolic and microenvironmental management strategies warrant further investigation as potential additions to standard oncologic management to improve disease control and quality of life in patients facing “palliative-only” situations. We encourage the design of prospective clinical studies and real-world registries to systematize this approach, refine patient selection, and optimize protocol components. Until such data are available, this case, together with our prior two publications, offers a hopeful message to clinicians and patients alike: even in advanced breast cancer, a precisely structured, metabolic oncology paradigm may unlock outcomes that exceed conventional expectations.

## Data Availability

The datasets presented in this article are not readily available because of ethical and privacy restrictions. Requests to access the datasets should be directed to the corresponding author.
